# Chemico-biological evaluation of carpachromene against key antimicrobial protein targets: an integrated *in-silico, in-vitro* approach for mechanistic insights

**DOI:** 10.3389/fphar.2026.1785267

**Published:** 2026-04-17

**Authors:** Aarif Nazir, Ibraq Khurshid, Shaista Masarat, Mushtaq A. Mir, Nasreena Bashir, Masood Ahmad Rizvi, Ishteyaq Majeed Shah, Venkatramanan Varadharajan, Fayaz Ahmad, Basharat Ahmad Bhat

**Affiliations:** 1 Advanced Research Lab, Department of Zoology, University of Kashmir, Jammu and Kashmir, India; 2 Department of Zoology, Central University of Kashmir, Ganderbal Kashmir, India; 3 Hydrobiology Lab, S.P College, Cluster University of Srinagar, Jammu and Kashmir, India; 4 Department of Clinical Laboratory, College of Applied Medical Sciences, King Khalid University, Abha, Saudi Arabia; 5 Department of Chemistry, School of Physical and Mathematical Sciences, University of Kashmir, Jammu and Kashmir, India; 6 Department of Biotechnology, PSG College of Technology, Coimbatore, India; 7 Department of Bio-Resources, School of Biological Sciences, University of Kashmir, Jammu and Kashmir, India

**Keywords:** antimicrobial activity, carpachromene, molecular docking, molecular dynamics simulation, supradecoration, Verbascum thapsus

## Abstract

**Ethnopharmacological Relevance:**

*Verbascum thapsus* L. is a prized medicinal plant from the Kashmir Himalaya traditionally utilized to treat many ailments, yet its active metabolites against antimicrobial mechanisms remain unclear.

**Aim of the Study:**

This work envisages an integrated *in-silico* and *in-vitro* approach to mechanistically investigate broad-spectrum antimicrobial activity of carpachromene, a supradecorated phytochemical from *V*. *thapsus*.

**Materials and Methods:**

Carpachromene was isolated through cold extraction from *V*. *thapsus*, followed by silica gel column chromatography with an optimized polarity solvent system, yielding a whitish amorphous solid confirmed by XRD, FTIR, ^1^H NMR and ^13^C NMR spectroscopy. *In-silico* analyses encompassed molecular docking of carpachromene against key microbial drug targets like sterol 14-α demethylase (CYP51), Dihydropteroate synthase (DHPS), GyrB ATPase domain, and Penicillin-Binding Protein 1 (PBP-1) followed by 100 ns molecular dynamics simulations assessing RMSD, RMSF, dynamic cross-correlation matrix (DCCM), principal component analysis (PCA), radius of gyration (Rg), and solvent accessible surface area (SASA). *In-vitro* antimicrobial activity was assessed using the agar well-diffusion method against clinical isolates: bacterial pathogens (*Escherichia coli* OP268610, *Staphylococcus aureus* OP268597, *Salmonella enterica* OP268585, *Pseudomonas aeruginosa* OP268614, *Klebsiella pneumoniae* OP268611, *Bacillus cereus* OP268602) and fungal pathogens (*Aspergillus niger* MTCC183, *A. fumigatus* MTCC282, *Candida albicans* MTCC343), evaluating concentration-dependent inhibition zones relative to standard controls (ciprofloxacin for bacteria; fluconazole for fungi).

**Results:**

Docking studies revealed robust binding affinities for carpachromene, ranging from −9.3 to −10.5 kcal/mol across targets (CYP51: −10.5 kcal/mol; *E. coli* DHPS: −9.6 kcal/mol; *S. aureus* GyrB: −9.4 kcal/mol; PBP-1: −9.3 kcal/mol) driven by hydrogen bonding (e.g., with active-site residues like Asp 73, Thr 165, Gly 77 in GyrB ATPase) and other hydrophobic interactions. MD simulations affirmed complex stability (RMSD: 1.2–1.5 Å; RMSF: 0.69–1.07 Å), with persistent intermolecular contacts, and coordinated residue motions via DCCM/PCA. *In-vitro* results revealed potent, dose-dependent activity, yielding maximum inhibition zones of 21 mm against *S. enterica* (50 μg/mL) and 10 mm against *C. albicans*.

**Conclusion:**

The mechanistic insights from computational analysis corroborated with *in-vitro* observations, highlighting carpachromene as a promising multi-target antimicrobial scaffold. These findings support supradecoration strategies for advancing carpachromene toward novel drug development against antimicrobial resistance.

## Graphical Abstract

**Figure:**
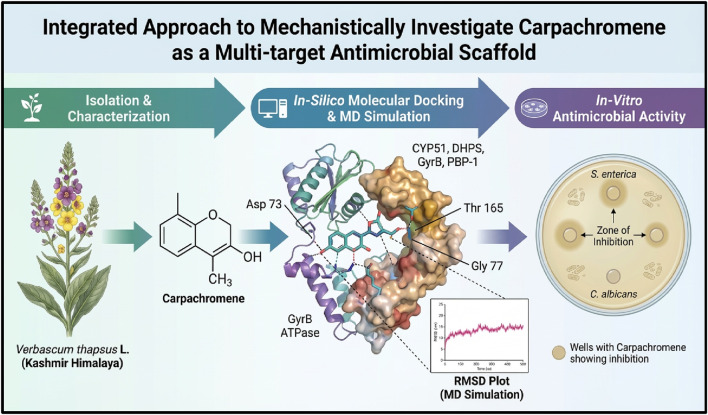
Carpachromene, a supradecorated flavonoid isolated from *Verbascum thapsus*, exhibits broad-spectrum antimicrobial activity with strong binding against key bacterial and fungal drug targets (CYP51, GyrB, DHPS, PBP-1) confirmed by molecular docking and stable dynamics simulations, highlighting its potential as a novel antimicrobial agent.

## Introduction

The escalating crisis of antimicrobial resistance (AMR) poses a severe threat to global public health, linked to millions of deaths annually and indicating a potential rise to 10 million deaths per year by 2050 if unchecked (Hamdani et al., 2020; Sheikh et al., 2022a; Sheikh et al., 2022b; Bhat et al., 2023; Naghavi et al., 2024; Ahmed et al., 2024; Ferraz, 2024). This alarming projection, representing a roughly 70% increase in attributable deaths compared to recent years, underscores the urgent need for innovative, sustainable antimicrobial agents to combat multidrug-resistant pathogens, including those in the ESKAPE group (SeyedAlinaghi et al., 2025).

Medicinal plants have long served as a rich reservoir of bioactive compounds, such as alkaloids, flavonoids, terpenoids, and phenolic derivatives, which exhibit potent antimicrobial properties through mechanisms including membrane disruption, efflux pump inhibition, quorum sensing interference, and biofilm suppression, offering promising alternatives or adjuncts to conventional antibiotics with often lower propensity for inducing resistance due to multi-target actions and synergistic effects (Mir et al., 2022a; Tabasum et al., 2022; Mir et al., 2022b; Bhat et al., 2022a; Bhat et al., 2022b). Recent systematic reviews and studies emphasize that plant-derived phytochemicals effectively target resistant strains, including ESKAPE pathogens, and demonstrate enhanced activity when combined with existing drugs, positioning natural products as vital sources for addressing AMR amid limited new antibiotic development (Angelini, 2024; El-Saadony et al., 2025; Saini et al., 2025; Tabasum et al., 2025).

*In-silico* approaches, particularly molecular docking and molecular dynamics (MD) simulations, have become indispensable in natural products-based antimicrobial drug discovery by providing detailed insights into ligand-protein interactions, binding affinities, complex stability, conformational dynamics, and mechanistic validation at the atomic level, thereby guiding the prioritization, optimization, and rational design of lead compounds from medicinal plants (Morris and Lim-Wilby, 2008; Meng et al., 2011; Fan et al., 2019; Aghajani et al., 2022; Saifi et al., 2024; Akter et al., 2024; Paggi et al., 2024; Xu et al., 2025). These computational tools enable efficient virtual screening of vast phytochemical libraries, prediction of multi-target engagement against key microbial enzymes and proteins, and assessment of binding stability over extended simulation timescales, significantly accelerating the transition from natural source identification to experimental validation while reducing costs and experimental failures compared to traditional high-throughput screening (Bhat et al., 2025).

Natural compounds remain a vital source of unique bioactive scaffolds, contributing up to 50% of approved drugs (Gani et al., 2026; Bhat et al., 2025a; Bhat et al., 2025b). Chromene derivatives, in particular, exhibit multifaceted pharmacological activities, including antimicrobial effects, positioning them as promising templates for combating drug resistance (Mohsin et al., 2020; Kaishap et al., 2025; Dar et al., 2025).

*Verbascum thapsus* L. (Scrophulariaceae), commonly known as mullein, is an ubiquitous herb valued for its various pharmacological properties (Alaaeldin et al., 2021; Jan et al., 2022; Hassan et al., 2020). Phytochemical profiling of its leaf extracts has revealed a rich array of compounds, largely flavonoids (Nadeem et al., 2021). In continuation of our research on Himalayan medicinal plants for exploration of value added phytochemicals, we have identified carpachromene among other bioactives in ethanolic leaf extract of *V. thapsus* (Nazir et al., 2025). An initial virtual screening via molecular docking prioritized this prenylated chromene derivative for its significant binding to essential microbial targets: sterol 14-α demethylase (CYP51), Dihydropteroate synthase, GyrB ATPase domain, and penicillin-binding protein-1.

Guided by these computational predictions, this study focused on targeted isolation of carpachromene from *V. thapsus* leaves, with structural confirmation via, XRD, FTIR, ^1^H and ^13^C NMR. Comprehensive *in-silico* and *in-vitro* antimicrobial evaluation, elucidated its multi-target mechanistic profile in antimicrobial drug development from sustainable natural sources.

## Materials and methods

### Chemicals

TLC grade silica gel coated aluminum sheet, Column grade silica gel (Silica gel 60, mesh size 0.063–0.200 mm), Dichloromethane (HPLC grade), n-Hexane (HPLC grade), Ethyl acetate (HPLC grade), Methanol (HPLC grade), n-Butanol (HPLC grade); The solvents used for extraction, chromatographic separation, and analytical TLC were obtained from Fisher Scientific India Ltd. and stored at room temperature. For routine use, they were transferred to 500 mL solvent bottles. Deuterated solvents (CDCl_3_, DMSO-d_6_, CD_3_OD, and acetone-d_6_, 99.9% purity) were purchased from Sigma-Aldrich India Ltd. and utilized for NMR analysis.

### Plant material

The leaves of *Verbascum thapsus* L. were collected from different locations in the Kashmir region during the summer of 2024 to ensure a representative sample. The specific collection sites, along with their geographical coordinates and altitudes, are as follows: Rambiara, Shopian (33°39′N, 74°39′E, 2000 m a.s.l.) on June 15, 2024; Keller, Shopian (33°73′N, 74°80′E, 2057 m a.s.l.) on July 5, 2024; Kathohalan, Shopian (33°78′N, 74°80′E, 2050 m a.s.l.) on July 20, 2024; and Aharbal, Kulgam (33°38′N, 74°46′E, 2266 m a.s.l.) on July 30, 2024.The plant material was identified and authenticated at the Centre for Biodiversity and Taxonomy, Department of Botany, University of Kashmir. Voucher specimens accession number: 9877-KASH.

### Plant extraction

Approximately 500 gms of plant material was subjected to cold extraction with solvents of increasing polarity (n-hexane, ethyl acetate, and ethanol). Extracts were concentrated under vacuum at 40 °C using a rotary evaporator. Hexane and ethyl acetate extracts were stored at −20 °C until analysis. The ethanolic extract was re-dissolved in 2.5% ethanol in distilled water and partitioned sequentially with dichloromethane and n-butanol, yielding three phases. Dichloromethane and n-butanol phases were dried over anhydrous sodium sulfate and evaporated, while the aqueous phase was freeze-dried. All dried fractions were stored at −20 °C in glass containers.

### Thin layer chromatography

Thin-layer chromatography (TLC) was employed to screen plant material for the presence of compounds.

### Column chromatography

Optimize the elution system for column chromatography and monitor column fractions. Plant extracts, fractions, or pure compounds were dissolved in solvents and applied as narrow bands approximately 1 cm above the TLC plate’s lower edge to enhance visualization and minimize compound overlap. Mobile phases consisted of n-hexane/dichloromethane, n-hexane/ethyl acetate, or ethyl acetate/methanol, selected based on sample polarity. Plates were examined under UV light at 254 nm (for aromatic compounds) and 366 nm (for conjugated double bonds).

**FIGURE 1:**
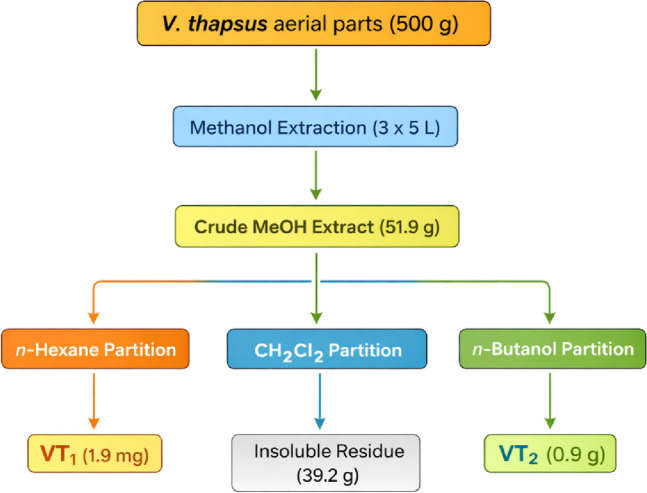
Extraction and fractionization of carpachromene compound from *V*. *thapsu*s Column chromatography (CC), Thin-layer chromatography (TLC), Gel filtration chromatography (GF), *Verbascum thapsus* (VT).

Open column chromatography (CC) was performed using silica gel 60 (mesh size 0.063–0.200 mm) with the wet packing technique. Silica gel was suspended in the least polar solvent of the elution system, poured into a glass chromatography column, and packed while eliminating air bubbles. Excess solvent was drained, allowing the column to settle. Samples were dissolved in a suitable solvent, adsorbed onto silica gel, and loaded onto the column. A cotton plug or filter paper was placed over the sample to prevent disturbance during elution, which was conducted either isocratically or with a gradient. Fractions were analyzed by TLC and pooled based on chemical profiles Figure 1.

### Isolation protocol

The column was initially eluted with 100% n-hexane, followed by a gradual increase in ethyl acetate concentration, and subsequently with increasing ethanol. Each solvent mixture (500 mL) was applied twice. The column dimensions were 7 cm in diameter and 15 cm in height. For gel filtration chromatography, GF1 was eluted with 50% dichloromethane in methanol, collecting approximately 2 mL per fraction, using a column measuring 2 cm in diameter and 100 cm in height. GF2 was eluted with 100% methanol under the same conditions, with approximately 2 mL collected per fraction. TLC: pooling fractions with similar chromatographic signatures.

### Structure elucidation

#### X-ray diffraction

The crystalline structure of compound was analysed using X-ray diffraction (XRD) with a Bruker D8 ADVANCE ECO diffractometer employing Cu Kα radiation (λ = 1.54060 Å). The X-ray source was operated at 30 kV and 25 mA. Diffraction data were recorded in continuous scan mode over a 2θ range of 32°–77° at a scan rate of 0.040° s^−1^.

The average crystallite size was estimated using the Debye–Scherrer equation,

$$D=K\lambda /\left(\text{Bcos }\theta \right)$$

where K = 0.9, λ is the X-ray wavelength, B is the full width at half maximum (FWHM) of the diffraction peak, and θ is the Bragg angle. The crystallite size was calculated from the major diffraction peaks.

#### Fourier Transform Infrared (FTIR) spectroscopy

FTIR spectrum of the material was recorded using a Shimadzu FTIR-8400 spectrophotometer (Shimadzu, Tokyo, Japan) with a spectral resolution of 4 cm^−1^ over the wavenumber range of 500–4000 cm^−1^. The sample was prepared as pellets by blending the material with potassium bromide (KBr) as a binding agent to ensure uniformity and facilitate optimal transmission of infrared radiation. The FTIR analysis provided detailed information on the functional groups and bonding characteristics within the material.

#### Nuclear magnetic resonance (NMR) spectrometry

NMR spectroscopy was performed using JEOL JNM-ECA 500 Spectrometer. Sample was dissolved in DMSO (^1^H δ 2.50 and ^13^C δ 39.52) and placed into NMR tube to a sample depth of 4 cm and the ^1^H (500 MHz) and ^13^C (125 MHz) spectra were measured. Chemical shifts were reported as δ units (ppm) with tetramethysilane (TMS) as internal standard and coupling constants (J) in Hz. Integration of the ^1^H NMR and ^13^C NMR data was performed by using DELTA version 5.0.4 software by JEOL.

### Molecular docking

#### Protein preparation

**FIGURE 2:**
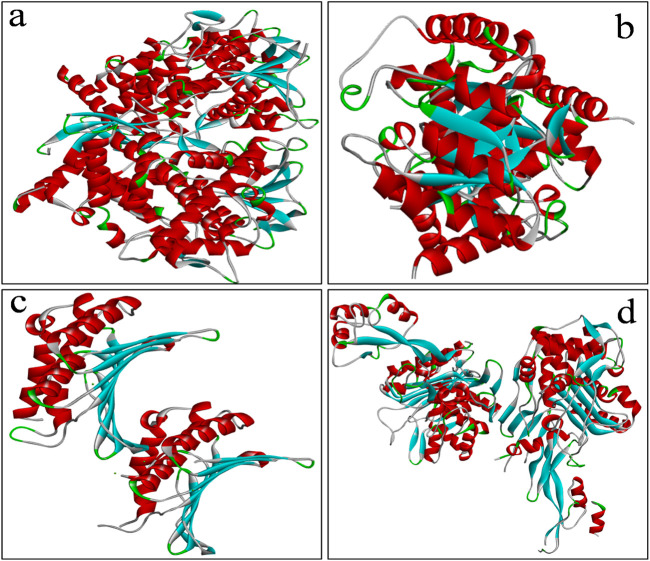
Crystal structures of the selected microbial target proteins viz; **(a)** sterol 14-alpha demethylase (CYP51), **(b)** *E. coli* (DHPS), **(c)** *S. aureus* GyrB ATPase domain, **(d)** 1 (PBP-1) retrieved from Protein Data Bank (PDB).

The crystal structures of the selected microbial target proteins viz;sterol 14-alpha demethylase (CYP51) (Pdb id: 5TZ1) from *C.albicans*, *E. coli* dihydropteroate synthase (DHPS) (Pdb id: 5V7A), *E. coli Gyrase B* (Pdb id: 1KZN), and Penicillin-Binding Protein-1 (PBP-1) (Pdb id: 7O4B) from *S*. *aureus* were retrieved from the Protein Data Bank (PDB) (Figure 2) (Berman et al., 2002). Protein preparation for molecular docking was performed using AutoDock tools (Morris et al., 2008) by removing extra chains, water molecules, adding hydrogen atoms, assigning Kollman charges, and converting the structures to Pdbqt format. Active sites were identified using the MOE software (Vilar et al., 2008). For docking studies performed with AutoDock vina (Morris et al., 2008), the grid box dimensions for each target protein were defined and recorded in a configuration file.

#### Ligand preparation and refinement

The bioactive ligand carpachromene was obtained from the PubChem database (Kim et al., 2016) in 3D Standard Data Format (SDF). Ligand preparation was conducted in AutoDock Tools, where Gasteiger charges were assigned, non-polar hydrogen atoms were merged, and rotational interactions were identified and modified.

#### Molecular docking analysis

Molecular docking analysis of carpachromene was performed using AutoDock Vina 4.0 (Trott and Olson, 2010), with the standard scripting method. Both target proteins and ligand were prepared in *.pdbqt* format after merging non-polar hydrogens. The docking process followed the Lamarckian Genetic Algorithm (LGA) and other docking standard procedures. LigPlot^+^ software (Laskowski and Swindells, 2011) was used to analyze ligand interactions, docked poses, and key amino acid residues involved in binding.

### Molecular dynamic simulation analysis

Docking simulations were performed using Desmond Schrödinger v3.8 (Schrödinger LLC, 2019; Jorgensen et al., 1996) to identify the compound with the best binding affinity. The simulations were conducted under the NPT ensemble conditions, maintaining a temperature of 300 K and pressure of 1 bar throughout the simulations, which were run over several nanoseconds. The OPLS_2005 force field (Kaminski et al., 2001) was applied to model the selected compound, and electrostatic interactions were treated using the Ewald summation method. Trajectories were sampled at 4.0 picosecond intervals to enhance the accuracy of the results. Ligand-protein interactions were analyzed using the Simulation Interaction Diagrams (SID) tool within the Desmond package. The stability of the ligand-protein complex was assessed by monitoring the Root Mean Square Deviation (RMSD), Root Mean Square Fluctuation (RMSF), Number of hydrogen bonds, Radius of gyrations, Solvent-Accessible Surface Area (SASA) of the complex over the course of the simulations, providing insights into the structural dynamics and binding stability.

### Antimicrobial activity

#### Procurement of microorganisms

The microbial strains used in the antimicrobial tests were obtained from two groups of clinical isolates 1) Bacterial pathogens: *E*. *coli* (Accession no: OP268610), *S*. *aureus* (Accession no: OP268597), *S*. *enterica* (Accession no: OP268585), *P*. *aeruginosa* (Accession no: OP268614) and *K*. *pneumoniae* (Accession no: OP268611) and *B*. *cereus* (Accession no: OP268602) and 2) Fungal pathogens: *A*. *niger* (MTCC183), *A*. *fumigatus* (MTCC282), *C*. *albicans* (MTCC343).The organisms were identified using standard microbiological techniques, followed by sub-culturing and isolation of pure cultures on nutrient agar media at 37 °C ± 0.5. Bacterial strains were procured from the Advanced Research Laboratory, Department of Zoology, University of Kashmir, Srinagar and fungal strains from IMTECH, Chandigarh.

The antimicrobial activity of carpachromene was evaluated against selected bacterial and fungal strains using the agar well-diffusion method. Mueller-Hinton agar (MHA) was prepared for bacterial cultures and Sabouraud dextrose agar (SDA) for fungal cultures according to Himedia specifications (38 g MHA and 65 g SDA per litre of distilled water). The media were autoclaved at 121 °C and 15 psi for 15 min (Gentek India Pvt. Ltd.), then aseptically poured (about 30 mL per plate) into sterile glass Petri dishes inside a laminar airflow cabinet (Toshiba, India) and allowed to solidify. Bacterial and fungal inoculum were evenly spread on the solidified agar surfaces with sterile cotton swabs. After a 10-min absorption period, five wells were made in each plate with a sterile cork borer. Each well was loaded with 50 µL of test or control solution: a positive control (+) of ciprofloxacin (0.1 mg/mL) for bacteria and itraconazole (0.35 mg/mL) for fungi, a negative control (−) of methanol (15 µL), and three different concentrations of carpachromene. The carpachromene wells were designated as C3, C2, and C1, where C3 contained 50 µL of a 50 μg/mL solution (highest concentration), C2 contained 50 µL of a 25 μg/mL solution (medium concentration), and C1 contained 50 µL of a 12.5 μg/mL solution (lowest concentration). Plates were sealed with parafilm to allow diffusion of the samples into the medium and incubated at 37 °C for 24 h for bacterial cultures and at 25 °C for 5 days for fungal cultures. After incubation, clear zones of inhibition around the wells were measured in millimeters to determine antimicrobial activity.

### Statistical analysis

The statistical analyses were performed with meticulous rigour to ensure the robustness and validity of the findings, utilizing a range of packages within R (v4.4.0; R Core Team, 2022; R Core TeamR, 2020). Data normality was evaluated through the Shapiro-Wilk test to ascertain the appropriateness of applying parametric statistical methods. A two-way analysis of variance (ANOVA) was conducted to evaluate the statistically significant variations in antimicrobial activity of carpachromene at different concentrations against the pathogenic bacteria and fungi (Supplementary Tables S1, S2). Data visualizations, such as heatmaps, and boxplots were generated using the ggplot2 package, enabling a detailed and comprehensive interpretation of observations (Wickham, 2016).

## Results and discussion

### Characteristic of VT1 as carpachromene

VT1 was isolated from the ethanolic extract of *V. thapsus* as a whitish amorphous solid which was TLC examined to be a single compound and subjected to NMR spectral analysis.

### FTIR reveals phenolic O–H, aromatic rings, C–O stretching (ethers or phenols), aliphatic side groups

Fourier Transform Infrared (FTIR) spectroscopy is a robust analytical technique for determining chemical structures and functional groups in diverse substances. By measuring sample absorption of infrared light, FTIR produces a spectrum that represents the vibrational frequencies of chemical bonds. FTIR spectroscopy was employed to identify and characterize the functional groups in the ethanolic fraction (VT2). The FTIR spectrum of the unknown compound reveals several key absorption bands that provide insight into the functional groups present Supplementary Figure S1.

Based on the presence of Phenolic O–H, Conjugated ketone, Aromatic rings, C–O stretching (ethers or phenols), Aliphatic side groups the compound is likely a substituted chromene or chromone derivative, commonly found in natural products such as flavonoids or coumarin-like structures. These compounds often exhibit antioxidant or antimicrobial activity.

### XRD profile supports the formation of polycyclic or heteroaromatic natural product

The X-ray diffraction (XRD) pattern of the synthesized compound, recorded in the 2θ range of 5°–80°, exhibits multiple sharp and intense peaks, particularly at approximately 16° and 22°, indicating a highly crystalline nature. The presence of numerous well-defined reflections within the 10°–40° region suggests a complex and ordered molecular structure. The absence of a broad amorphous halo further confirms the crystalline purity of the material, ruling out significant amorphous content. These characteristics are typical of low-molecular-weight organic compounds with rigid aromatic or heterocyclic frameworks. The sharpness and intensity of the diffraction peaks imply a well-developed long-range order within the crystal lattice, which is consistent with a molecular system possessing strong intermolecular interactions, such as hydrogen bonding or π–π stacking. This XRD profile supports the formation of a structurally ordered organic compound, potentially belonging to a class of polycyclic or heteroaromatic natural products Supplementary Figure S2.

### NMR spectral analysis established the isolated compound as carpachromene

^1^H NMR (400 MHz, DMSO d_6_) δ 7.45–7.38 (m, 2H, Ar-Region), 6.96–6.82 (m, 2H, Ar-Region), 6.59 (1H, Doublet, Alkene), 6.30 (s, 1H, Alkene), 6.23 (s, 1H, Ar-Region), 6.17 (1H, Doublet, Alkene), 1.56 (s, 6H). (Rest all peaks are impurities and solvent DMSO at 2.5 ppm).

^13^C NMR (101 DMSO d6) δ 181.69 (s), 163.74 (s), 161.67 (s), 160.68 (s), 158.00 (s), 157.06 (s), 128.91–128.51 (m), 127.63 (s), 122.32 (s), 117.11 (s), 116.07–115.86 (m), 105.66 (d, J = 8.1 Hz), 104.57 (s), 93.95 (s), 77.27 (s), 27.82–27.51 (m) (Supplementary Figure S3).

### Molecular docking shows strong affinity between carpachromene and specific microbial drug target proteins

**FIGURE 3:**
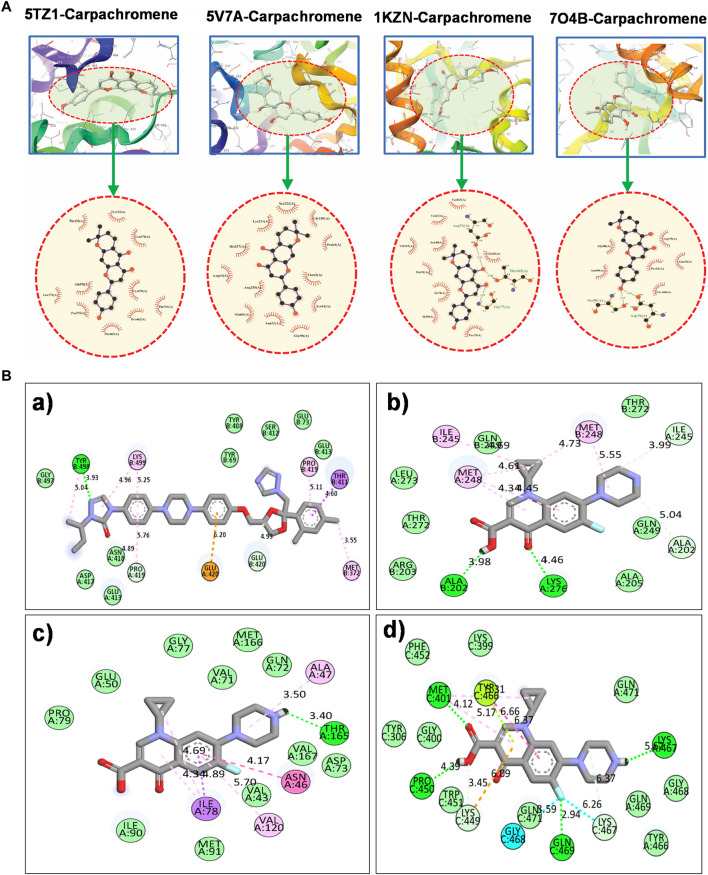
**(A)** The diagram illustrates molecular docking results, showing predicted binding poses of carpachromene in the active pockets of four antimicrobial target proteins. The top panels display 3D views with the protein in ribbon representation and carpachromene as sticks, highlighting key interactions within the circled binding sites. The bottom panels provide 2D ligand interaction maps, detailing hydrogen bonds (green lines), hydrophobic contacts, and π-π stacking with surrounding amino acid residues. **(B)** Comparative docking interaction maps of the reference drugs itraconazole (antifungal control) with **(a)** sterol 14-α-demethylase (CYP51) and ciprofloxacin (antibacterial control) with bacterial targets with **(b)** *E. coli* (DHPS), **(c)** *S. aureus* GyrB ATPase domain, **(d)** 1 (PBP-1) illustrating their interactions within the respective active sites.

The Figures 3A,B illustrates the computationally predicted binding conformations and key interactions of carpachromene a naturally occurring flavonoid isolated from *V. thapsus* known for its α-glucosidase inhibitory, anti-inflammatory, and insulin-sensitizing properties (Alaaeldin et al., 2021), and the control drugs within the active sites of six microbial drug target protein. These targets were selected from publicly available Protein Data Bank (PDB) structures, originally determined with distinct ligands, and repurposed here for molecular docking simulations to explore carpachromene’s potential polypharmacological profile. Docking into the protein-binding site of DHPS of *E. coli*, a validated antibacterial target inhibited by sulfonamides. The 3D poses positions carpachromene within the active cleft, forming H-bonds with Glu residues, alongside hydrophobic interactions, suggesting potential disruption of folate biosynthesis and antibacterial efficacy. Binding to the 24 kDa ATPase domain of DNA gyrase of *E. coli*, a topoisomerase target for agents such as coumarins. The 3D model shows carpachromene accommodated in the ATP-binding pocket, with the 2D diagram revealing H-bonds to Asp, Thr and Gly and additional contacts, indicative of competitive inhibition compared to clorobiocin. Interaction with the PBP-1 from *S*. *aureus*, an antibacterial hotspot for penicillin G-like inhibitors. Carpachromene occupies the penicillin G-binding region in the 3D view, engaging in H-bonds with Ser and Asp as detailed in the 2D map Table 1.

**TABLE 1:** Binding energies (kcal/mol) obtained from the docking calculations of the isolated compound (carpachromene) with microbial target proteins.

Compound	Protein targets	Binding energy (kcal/mol)	Hydrogen bonds	Other interactions
Carpachromene	Sterol 14-alpha demethylase (CYP51)	−10.54	__	TYR 132, LEU 376, THR 311, CYS 470, THR 341, PRO 462, PHE 463, PRO 375, LEU 370, ALA 476
	*E. coli* (DHPS)	−9.62	GLU 60	SER222, GLY 189, PRO 64, THR 62, SER 61, GLY 58, ASN 22, ARG 63, ARG255, HIS257, LYS 221
	*S. aureus* GyrB ATPase domain	−9.40	ASP 73, THR 165, GLY 77	VAL 167, VAL 43, ASN 46, VAL 120, MET 91, ILE 78, ILE 90, PRO 79, GLU 50
	PBP-1	−9.30	SER 50, ASP 76	GLY 80, ASN 49, THR 168, GLU 53, PRO 82, ARG 79
Ciprofloxacin (control)	DHPS	−6.30	ALA202, LYS276	ILE245, GLN249, MET248, THR272, LEU273, ARG203, ALA205
	GyrB	−6.59	THR165	GLY77, MET166, GLN72, VAL71, GLU50, PRO79, ALA47, VAL167, ASP73, ASN46, ILE78, VAL43, VAL120, ILE90, MET91
	PBP-1	−6.42	MET401, PRO450, GLN469, LYS467	PHE452, LYS399, TYR446, TYR306, GLY400, TRP451, LYS449, GLN471, GLY468, TYR466
Itraconazole (control)	CYP51	−8.30	TYR498	GLY497, LYS499, ASN419, PRO419, ASP417, GLU418, GLU411, TYR408, TYR69, SER412, GLU413, THR411, MET372, GLU420

The docking affinities observed for carpachromene (ranging from −9.3 kcal/mol for *S. aureus* PBP-1 to −10.5 kcal/mol for *C. albicans* CYP51) compare favorably with, and in most cases surpass, previously reported computational binding energies for this compound against other therapeutic targets. For instance, Rauf et al. (2022) demonstrated docking scores of −6.215 to −6.732 kcal/mol for carpachromene against urease, tyrosinase, and phosphodiesterase enzymes from Ficus benghalensis extracts, with key hydrogen-bond interactions mirroring those in our CYP51 and DHPS complexes (e.g., H-bonds with active-site Asp/Ser residues). Similarly, Ramalingam et al. (2023) identified carpachromene as a top-ranked inhibitor of KRAS G12D mutant (−8.64 kcal/mol), forming two hydrogen bonds (Leu6, Asp54) and hydrophobic contacts analogous to our π-stacking interactions with Phe residues in DHPS and GyrB. Our superior scores likely stem from optimized orientation within microbial pockets, driven by the chromene scaffold’s phenolic OH groups, which facilitate persistent H-bonding.

### Molecular dynamics (MD) simulation analysis

To assess the dynamic stability and interaction profiles of carpachromene, a chromene-based compound with potential pharmacological properties we performed 100 ns molecular dynamics (MD) simulations for its complexes with four key microbial protein targets: sterol 14α-demethylase (CYP51) from *C*. *albicans*, a fungal enzyme essential for ergosterol biosynthesis and a validated antifungal target; DHPS from *E*. *coli*, a bacterial folate pathway enzyme targeted by sulfonamide antibiotics; the 24 kDa ATPase domain of DNA gyrase B from *E. coli*, a topoisomerase subunit inhibited by coumarin-class antibiotics; and bacterial Penicillin-binding protein. These targets were selected to evaluate carpachromene’s broad-spectrum potential against fungal and bacterial pathogens.

### RMSD analysis

**FIGURE 4:**
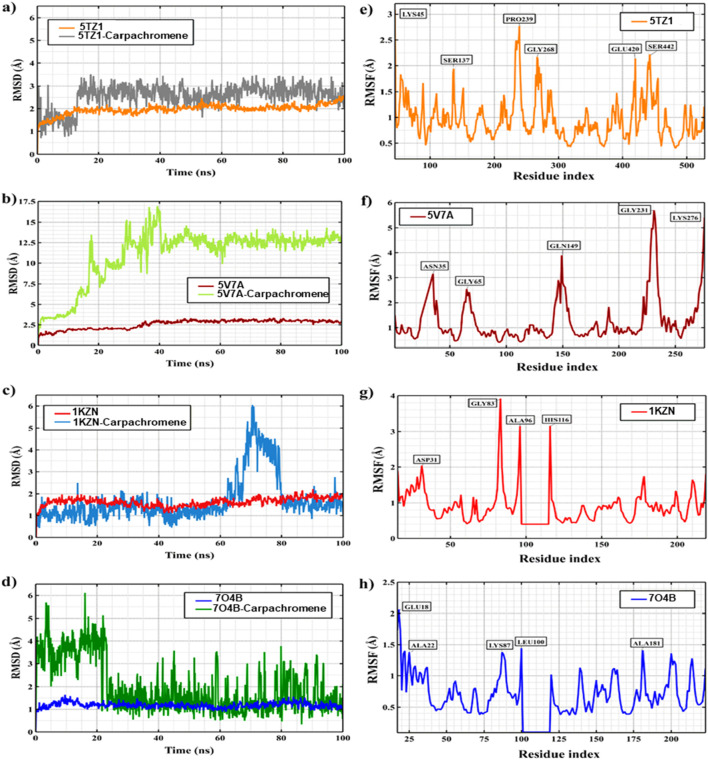
Root Mean Square Deviation (RMSD) and Root Mean Square Fluctuation (RMSF) analyses from 100 ns molecular dynamics simulations of carpachromene complexes with four antimicrobial target proteins. **(a–d)** show RMSD trajectories over time: protein Cα backbone and carpachromene ligand, indicating overall structural stability and ligand binding persistence in the active site. **(e–h)** display per-residue RMSF of the protein Cα atoms, with key fluctuating residues labeled, highlighting flexible regions (typically loops) versus rigid secondary structures. These results demonstrate stable carpachromene binding across simulations (low ligand RMSD in most cases) and identify residue flexibility, supporting the reliability of docked poses and potential multi-target antimicrobial activity of carpachromene.

The root-mean-square deviation (RMSD) of the protein Cα backbone and ligand heavy atoms was computed relative to the equilibrated starting structures at 0.1 ns intervals Figures 4a–d. All systems exhibited an initial rise in RMSD during the equilibration phase (0–10 ns), followed by convergence toward plateau regions, indicating stabilization of the trajectories without significant unfolding of the protein structures. The protein backbone RMSDs for most systems stabilized within ∼1.2–1.5 Å, reflecting the structural rigidity and integrity typical of well-folded proteins during molecular dynamics simulations. The ligand RMSDs displayed system-specific behavior. The 1KZN_carpachromene and 5TZ1_carpachromene complexes maintained relatively low ligand deviations (∼1.0–1.6 Å), suggesting stable binding conformations with minimal displacement within the active site. In contrast, the 7O4B_carpachromene complex exhibited moderately higher fluctuations (up to ∼3.5 Å on average), which may be attributed to a relatively solvent-accessible binding environment or local flexibility within the binding pocket. Notably, the 5V7A–carpachromene complex showed a pronounced RMSD increase compared with the other systems, reaching values of approximately ∼16–17 Å during the early simulation phase and stabilizing around ∼12–14 Å after ∼40 ns. This elevated deviation likely reflects ligand-induced conformational rearrangements within the protein structure, potentially associated with adaptive movements of flexible loops or domain regions surrounding the binding pocket. Importantly, the RMSD profile eventually reaches a relatively stable plateau, suggesting that the system attains a new conformational equilibrium rather than undergoing progressive structural destabilization. Such behavior is consistent with induced-fit binding mechanisms frequently observed in protein–ligand complexes. Quantitative summaries are provided in Table 2, confirming the reproducibility of these trends (standard deviations <0.3 Å for backbones, <0.8 Å for ligands).

**TABLE 2:** Average RMSD (± standard deviation) over the 100 ns trajectories for protein Cα backbones and ligand heavy atoms. Values were calculated excluding the first 10 ns equilibration phase.

Protein IDs	Protein backbone RMSD (Å)	Ligand RMSD (Å)
1KZN	1.52 ± 0.27	1.02 ± 0.30
7O4B	1.18 ± 0.21	3.52 ± 0.81
5TZ1	1.49 ± 0.24	1.55 ± 0.47
5V7A	1.50 ± 0.26	3.32 ± 0.52

### RMSF analysis

Residue-wise root-mean-square fluctuations (RMSF) of the Cα atoms were calculated to evaluate the local flexibility of residues in the presence of the carpachromene ligand during the molecular dynamics simulations Figures 4e–h. The average RMSF values ranged from 0.69 Å for the 7O4B_carpachromene complex to 1.07 Å for the 5TZ1_carpachromene complex, indicating overall structural compactness and limited global conformational motion upon ligand binding. Regions exhibiting low RMSF values (<0.5 Å) were predominantly associated with stable secondary structural elements such as α-helices and β-sheets, suggesting that the core architecture of the proteins remained largely preserved in the presence of carpachromene. In contrast, higher RMSF peaks were primarily observed in loop regions and terminal residues, which are inherently flexible and may contribute to local conformational adjustments required for ligand accommodation within the binding pockets.

### Notable high-fluctuation residues (>3 Å) include

The residue-level fluctuation analysis further identified several flexibility hotspots across the protein–carpachromene complexes. In the 1KZN_carpachromene system, Gly83 (3.92 Å) exhibited the highest fluctuation, likely representing a solvent-exposed loop region contributing to hinge-like conformational motions. For the 7O4B_carpachromene complex, Glu18 (2.06 Å) near the N-terminus displayed moderate flexibility, which may facilitate adaptive gating and transient conformational adjustments upon ligand binding. In the 5TZ1_carpachromene complex, Lys45 (3.07 Å) showed increased mobility within a putative regulatory loop that could influence ligand accessibility to the binding pocket. Similarly, the 5V7A_carpachromene complex revealed notable fluctuation at Asn35 (3.15 Å), located near the binding interface, suggesting the presence of dynamic hydrogen-bonding interactions and local structural rearrangements associated with ligand accommodation. These flexibility hotspots (Table 3) correspond to regions that are often structurally unresolved or weakly defined in crystallographic models, indicating inherently dynamic segments of the proteins. Such regions may play important roles in allosteric regulation, ligand recognition, and entropy-driven binding affinity, particularly in protein–ligand systems involving flexible natural compounds such as carpachromene.

**TABLE 3:** Summary of Cα RMSF statistics across all residues. Averages and standard deviations are trajectory-wide; maxima identify the most flexible sites.

Docked complexes	Average RMSF (Å)	Std. Dev. (Å)	Max RMSF (Å)	Residue with max RMSF
1KZN_carpachromene	0.961	0.585	3.921	Gly83
7O4B_carpachromene	0.687	0.403	2.057	Glu18
5TZ1_carpachromene	1.074	0.368	2.75	Pro239
5V7A_carpachromene	0.976	0.513	3.147	Asn35

The combined RMSD and RMSF analyses provide insights into the dynamic stability of the protein–carpachromene complexes. The 1KZN_carpachromene and 5TZ1_carpachromene systems exhibited comparatively lower structural deviations and residue fluctuations, indicating the formation of relatively rigid complexes that may favor high-affinity and shape-complementary binding interactions. In contrast, the 7O4B_carpachromene and 5V7A_carpachromene complexes demonstrated higher dynamic behavior, suggesting a greater degree of conformational adaptability within their binding environments.

### Radius of gyration (Rg) analysis

**FIGURE 5:**
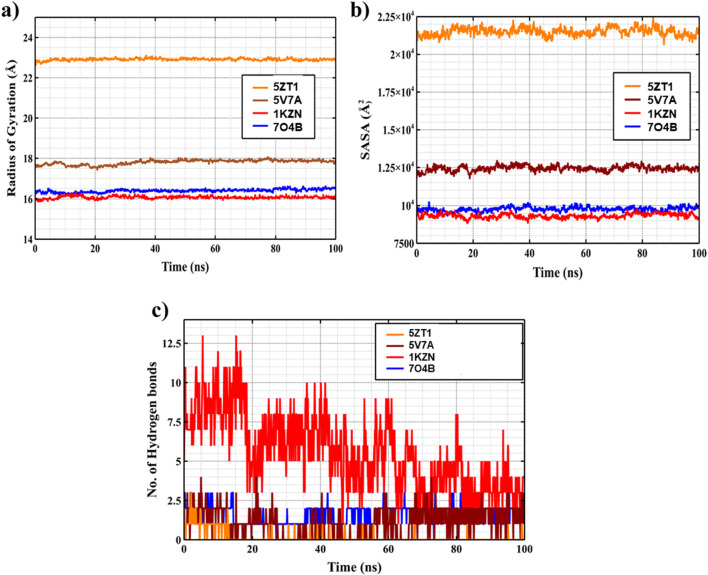
Time evolution of key structural properties during 100 ns molecular dynamics simulations of carpachromene complexes with four antimicrobial target proteins. **(a)** shows the radius of gyration (Rg) of the protein Cα atoms, with all complexes maintaining stable compactness (Rg values ∼16–23 Å) throughout the simulation, indicating no significant unfolding. **(b)** displays the solvent-accessible surface area (SASA), revealing consistent exposure patterns with minor fluctuations, suggesting preserved overall protein conformation and ligand shielding in the binding pockets. **(c)** illustrates the number of intermolecular hydrogen bonds between carpachromene and each protein, with the 1KZN_ carpachromene complex (red) forming the highest and most persistent bonds, while others show fewer but sustained interactions, supporting binding stability across targets.

The radius of gyration (Rg), a measure of protein compactness, remained largely invariant across all structures Figure 5a. For 1KZN, 7O4B, and 5V7A, Rg values stabilized around 16 Å, 16.3 Å, and 17.8 Å, respectively, with fluctuations <0.5 Å, suggesting maintenance of a compact, native-like fold. The larger 5TZ1 structure exhibited a higher average Rg of ∼22.9 Å, consistent with its extended architecture, but similarly showed negligible drift (<0.3 Å standard deviation), underscoring global structural integrity without expansion or collapse.

### Solvent-accessible surface area (SASA)analysis

Solvent-accessible surface area (SASA) profiles further supported solvent equilibration and hydrophobic core preservation Figure 5b. Average SASA values were 9300 ± 150 Å^2^ for 1KZN, 9800 ± 200 Å^2^ for 7O4B, 21200 ± 300 Å^2^ for 5TZ1, and 12300 ± 250 Å^2^ for 5V7A, with time-dependent oscillations reflecting transient side-chain dynamics but no systematic trends toward burial or exposure. These steady-state exposures align with expected values for folded proteins of comparable size, implying effective solvation without aggregation or denaturation.

### Intra-molecular hydrogen bonds (H-bonds) analysis

The distribution of intra-molecular hydrogen bonds (H-bonds) delineated unimodal distributions anchored at biologically pertinent stoichiometries Figure 5c. 1KZN maintained ∼8 H-bonds on average (range: 4–13), 7O4B ∼2 (range: 1–3), 5TZ1 ∼1 (range: 0–3), and 5V7A ∼1–2 (range: 0–4), with the histogram peaking at these medians and exhibiting Poisson-like tails from local fluctuations. The absence of bimodal distributions or shifts toward zero bonds indicates preserved secondary elements, such as α-helices and β-sheets, critical for functional stability.

### Protein-ligand (P-L) contacts

**FIGURE 6:**
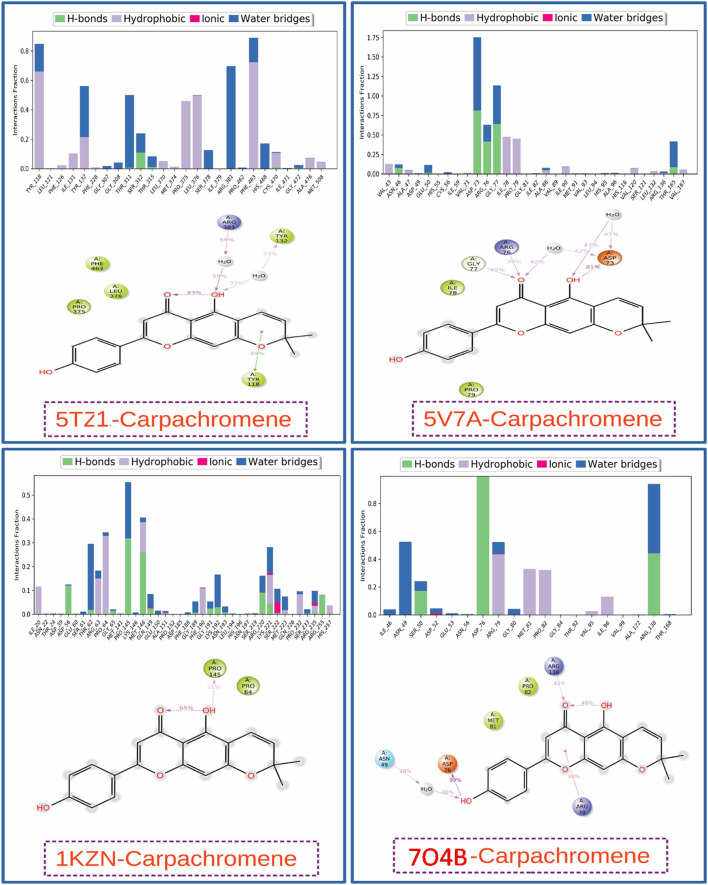
The figure presents protein-ligand interaction profiles from the 100 ns simulation as stacked bar charts and corresponding 2D interaction diagrams for the complexes: 5TZ1_carpachromene; 5V7A_ carpachromene; 1KZN_carpachromene; 7O4B_carpachromene. Protein-ligand interaction profiles of carpachromene complexes with four antimicrobial targets derived from 100 ns molecular dynamics simulations. Each panel combines a stacked bar chart showing the fraction of simulation frames (0–1.0) in which specific interaction types persist (green: hydrogen bonds; light green: hydrophobic; purple: ionic; blue: water bridges) and a 2D diagram highlighting key interacting residues. The charts reveal varying interaction patterns: 5TZ1_carpachromene and 7O4B_carpachromene show strong water-mediated bridges and hydrophobic contacts, while 1KZN_carpachromene and 5V7A_carpachromene complexes exhibit prominent conventional hydrogen bonds alongside hydrophobic interactions. These persistent non-covalent interactions throughout the simulation confirm the stability of the docked Carpachromene poses and support its potential as a multi-target antimicrobial agent.

Time-dependent interaction profiles from 100 ns MD simulations of carpachromene bound to microbial targets. Figures 6a–d comprises (upper) stacked bar charts depicting the average number of interactions per frame across categories. H-bonds (green), hydrophobic contacts (blue), ionic bonds (purple), and water bridges (cyan)binned in 10 ns intervals along the x-axis (0–100 ns); and (lower) representative snapshots of the binding interface at ∼50 ns, with carpachromene in yellow licorice representation and key protein residues/interactions highlighted (green dashed lines: H-bonds; blue: hydrophobic; purple: ionic; cyan: water bridges). (a) 5TZ1_carpachromene complex, showing sustained H-bonding dominance; (b) 5V7A_carpachromene complex, with balanced hydrophobic and ionic contributions; (c) 1KZN_carpachromene complex, featuring variable H-bonds and hydrophobic stability; (d) 7O4B_carpachromene complex, exhibiting peak water-mediated interactions.

### Principal component analysis (PCA) analysis

**FIGURE 7:**
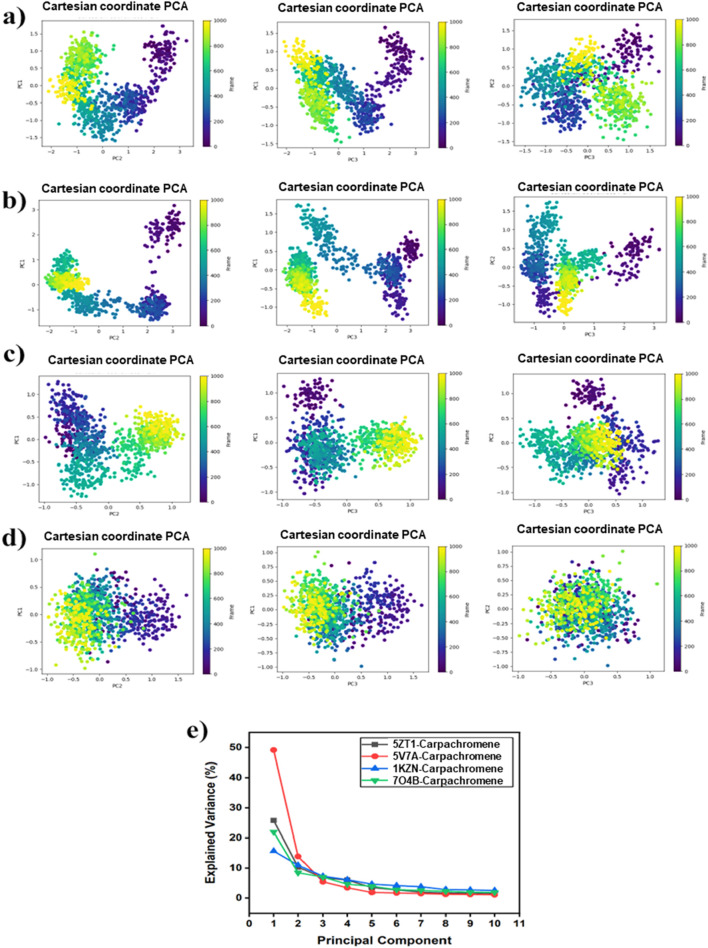
Principal Component Analysis (PCA) of conformational ensembles from 100 ns molecular dynamics simulations of carpachromene complexes with four antimicrobial target proteins: Each subpanel in **(a–d)** shows three 2D projections (PC1 vs. PC2, PC1 vs. PC3, PC2 vs. PC3) of the Cartesian PCA on protein Cα atoms, with points colored by simulation time, revealing the extent and distribution of sampled conformational space and the plots indicate varying degrees of conformational sampling. **(e)** presents the scree plot of eigenvalue-ranked explained variance percentage, demonstrating that the first few principal components capture the majority of motion upon carpachromene binding.

Principal Component Analysis (PCA) provides crucial insights into the dynamic behavior and internal motions of protein-ligand complexes during molecular dynamics (MD) simulations. In this study, PCA was employed to evaluate the collective atomic fluctuations of the complexes by plotting eigen values against the corresponding eigenvector indices (modes) for the top 10 components. These plots capture the dominant motion patterns of the system. The Figure 7a illustrates that the 5TZ1_carpachromene complex exhibited the highest eigenvalues among the top eigenvectors, indicating that these principal modes significantly contribute to the overall motion of the system. The first five eigenvectors alone accounted for 73.7% of the total variance, underscoring their critical role in driving conformational changes. A detailed analysis of principal components revealed that PC1 contributed 49.1%, PC2 contributed 13.9%, and PC3 contributed 5.4% of the motion, suggesting a stable and compact conformation for 5TZ1_carpachromene, as evidenced by its lower variance in PC3. Similarly, Figure 7b shows that the 5V7A_carpachromene complex exhibited variability across PC1 (25.8%), PC2 (10.3%), and PC3 (6.8%), indicating moderate distribution of motion across multiple components. Figure 7c presents the 7O4B_carpachromene complex, where motion was distributed as PC1 (21.9%), PC2 (8.4%), and PC3 (7.0%), reflecting a relatively more dispersed and flexible dynamic behavior. Figure 7d highlights that 1KZN_carpachromene exhibited relatively high variability in PC1 (15.7%) and moderate levels in PC2 (11.0%) and PC3 (7.3%). Color-coded graphs in Figures 7a–d represent mobility levels, where blue denotes high mobility, white indicates moderate motion, and red signifies limited flexibility, allowing for a visual comparison of the complexe’s dynamic profiles.

### Dynamic cross-correlation map (DCCM) analysis of carpachromene-docked protein complexes

**FIGURE 8:**
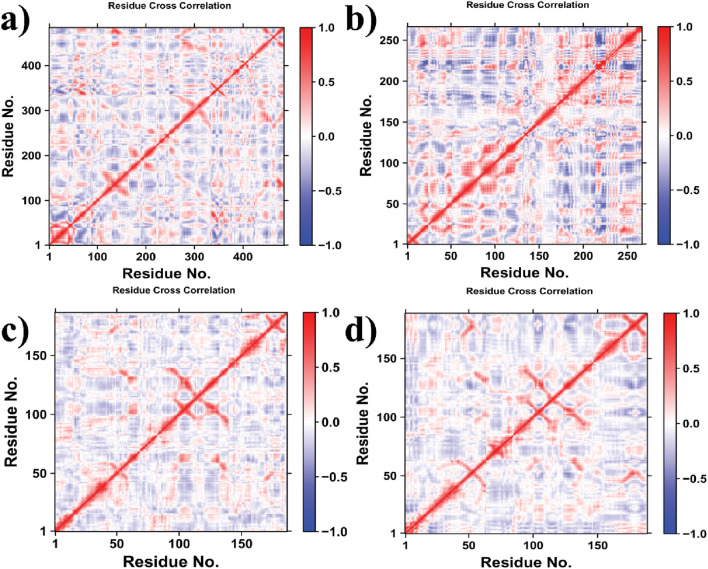
DCCM correlation maps showing interaction dynamics for the same complexes: **(a)** 5V7A_carpachromene, **(b)** 5TZ1_carpachromene, **(c)** 1KZN_carpachromene **(d)** 7O4B_carpachromene complex.

Dynamic cross-correlation maps (DCCMs) derived from molecular dynamics simulations elucidate residue fluctuation correlations in carpachromene-bound protein complexes Figure 8. Values range from +1 (concerted motion) to −1 (anti-correlation), with off-diagonal metrics assessing global dynamics (Table 4).

**TABLE 4:** Global DCCM Metrics of the docked complexes.

Complex	Residues	Mean correlation	Variance
5V7A_carpachromene	266	0.12	0.08
5TZ1_carpachromene	∼484	0.08	0.11
1KZN_carpachromene	186	0.15	0.07
7O4B_carpachromene	188	0.14	0.09

In the dynamic cross-correlation matrix (DCCM), positively correlated residue motions are represented in red, while negatively correlated (anti-correlated) motions are depicted in blue. The DCCM for the larger protein structure Figure 8a reveals extensive blocks of strong positive correlations along the diagonal and in the central regions (residues ∼100–300), indicative of coordinated intra-domain dynamics and potential allosteric communication pathways. In contrast, the DCCM for PDB ID: 5V7A Figure 8b displays more fragmented positive correlation clusters, particularly in the N- and C-terminal segments, with pronounced anti-correlations bridging secondary structure elements, suggesting greater structural flexibility and domain decoupling. For the smaller systems, the DCCM of PDB ID: 1KZN Figure 8c exhibits localized positive correlations in short-range residue pairs (e.g., residues 50–100), highlighting rigid, cooperative motions within compact motifs amid widespread anti-correlations elsewhere. Similarly, the DCCM for PDB ID: 7O4B Figure 8d shows concentrated positive correlations near the diagonal (residues ∼20–80 and ∼120–150), implying stable local interactions but limited long-range synchrony, consistent with a more modular architecture. Overall, these patterns underscore varying degrees of residue coupling across the structures, with implications for functional conformational changes.

### Field energy landscape (FEL) analysis

**FIGURE 9:**
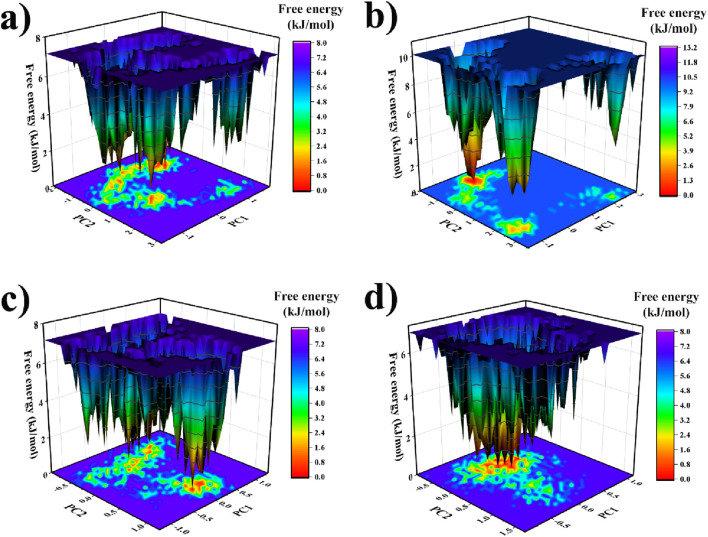
**(a-d)** Free energy landscapes (FELs) of protein-ligand complexes with carpachromene from 100 ns MD simulations, projected onto PC1 and PC2. Gibbs free energy (ΔG) is in kJ/mol; blue = stable minima, red = high-energy regions.

Free energy landscapes (FELs) were constructed by projecting the 100 ns MD trajectories onto the first two principal components (PC1 and PC2) to delineate the conformational ensembles and relative stabilities of the complexes Figure 9. The Gibbs free energy (ΔG) is expressed in kJ/mol, with lower values (blue regions) representing thermodynamically favored conformations and higher values (red regions) indicating less populated or metastable states. The FEL of the 1KZN complex (a) reveals a rugged landscape with multiple shallow and dispersed energy basins, spanning PC1 ≈ −1.2 to 1.3 Å and PC2 ≈ −1.0 to 1.2 Å, with a global minimum of approximately 0 kJ/mol near PC1 ≈ 0.0 Å and PC2 ≈ 0.8 Å. This profile indicates enhanced conformational flexibility, greater exploration of conformational space, and the presence of several metastable states, consistent with dynamic heterogeneity induced by the carpachromene ligand in the *E. coli* DNA gyrase B 24 kDa domain. In contrast, the FEL of the 7O4B_ carpachromene complex (b) exhibits a single, deep, and well-defined energy basin centered near PC1 ≈ 0.4 Å and PC2 ≈ −0.2 Å, with a global minimum of approximately 0 kJ/mol. The narrow and compact basin reflects restricted conformational sampling and high structural stability, aligning with strong binding of the carpachromene ligand to the PBP-1 from *S*. *aureus*.

The FEL of the 5TZ1_carpachromene complex (c) shows a broad and fragmented energy landscape, spanning PC1 ≈ −1.5 to 1.8 Å and PC2 ≈ −2.2 to 3.2 Å, with the global minimum near PC1 ≈ 0.9 Å and PC2 ≈ −0.6 Å. Multiple shallow minima and low energy barriers are evident, suggesting significant conformational diversity and plasticity in the sterol 14-alpha demethylase (CYP51) from *C*. *albicans* bound to the carpachromene ligand molecule. The FEL of the 5V7A_ carpachromene complex (d) displays an intermediate yet distinctly fragmented FEL, with a primary basin near PC1 ≈ 0.1 Å and PC2 ≈ −1.3 Å accompanied by elevated surrounding regions and higher energy barriers across PC1 ≈ −1.5 to 3.1 Å and PC2 ≈ −2.1 to 3.5 Å. The global minimum is approximately 0 kJ/mol, implying moderate destabilization and restricted yet less cohesive conformational sampling compared to the more stable complexes, in the context of *E. coli* DHPS complexed with the carpachromene compound. Across all targets, carpachromene promotes varying degrees of structural plasticity, with 7O4B_carpachromene exhibiting the greatest conformational restraint and 5TZ1_carpachromene the most extensive exploration. This suggests ligand-dependent modulation of dynamics, potentially linked to carpachromene’s multi-target inhibitory profile.

### Antimicrobial assay

**FIGURE 10:**
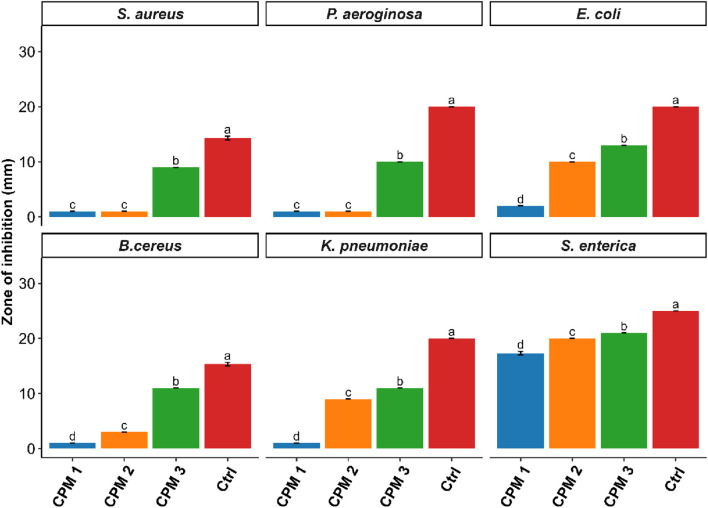
Quantitative assessment of antibacterial activity of CPM against various bacterial strains.

The antimicrobial efficacy of carpachromene was assessed against a broad spectrum of clinically relevant bacterial and fungal pathogens at concentrations of 12.5 μg mL^−1^ (CPM 1), 25 μg mL^−1^ (CPM 2), and 50 μg mL^−1^ (CPM 3), with results summarized in Supplementary Figure S4 and Supplementary Table S1, S2. A clear concentration-dependent increase in the zone of inhibition was evident across all tested organisms, confirming the potent bioactivity of carpachromene. Among the bacterial strains, CPM 3 produced the strongest effect against *S. enterica*, yielding a 21 mm inhibition zone that closely approached the 25 mm zone observed for the positive control, ciprofloxacin. This was followed by a 13.00 mm zone of inhibition against *E. coli*, while the remaining bacterial species exhibited moderate susceptibility. Even at the lowest concentration, CPM 1 elicited measurable inhibition of *S. enterica*, suggesting either an intrinsic vulnerability of this pathogen or a high bioavailability of the compound. In contrast, Gram-positive organisms such as *Staphylococcus aureus* and *B. cereus* required higher CPM concentrations for marked inhibition; nevertheless, statistically significant differences (p < 0.05) were observed among all treatment groups Figure 10.

**FIGURE 11:**
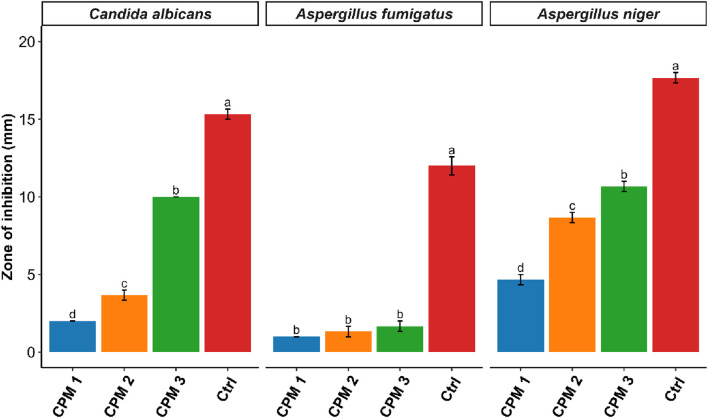
Quantitative evaluation of antifungal activity of CPM against fungal strains.

Similarly, in the antifungal assays, CPM 3 exhibited pronounced antifungal activity against *C. albicans* and *A. niger*, with inhibition zones approaching that of the positive antifungal control, highlighting its potential as a natural antifungal agent. *A. fumigatus*, however, displayed relative resistance, with minimal response to CPM treatment even at the highest concentration, possibly due to its robust spore wall or inherent enzymatic detoxification mechanisms. Statistical analysis confirmed that the increase in activity from CPM 1 to CPM 3 was significant in all responsive strains, as denoted by distinct superscript letters. The consistent pattern of increasing inhibition with concentration underscores the potency and dose-dependence of the formulation Figure 11.

**FIGURE 12:**
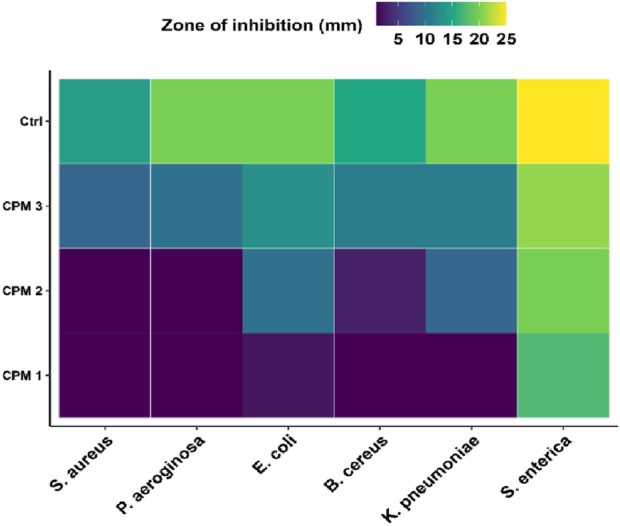
Heatmap visualization of antibacterial potency of CPM across multiple bacterial strains.

**FIGURE 13:**
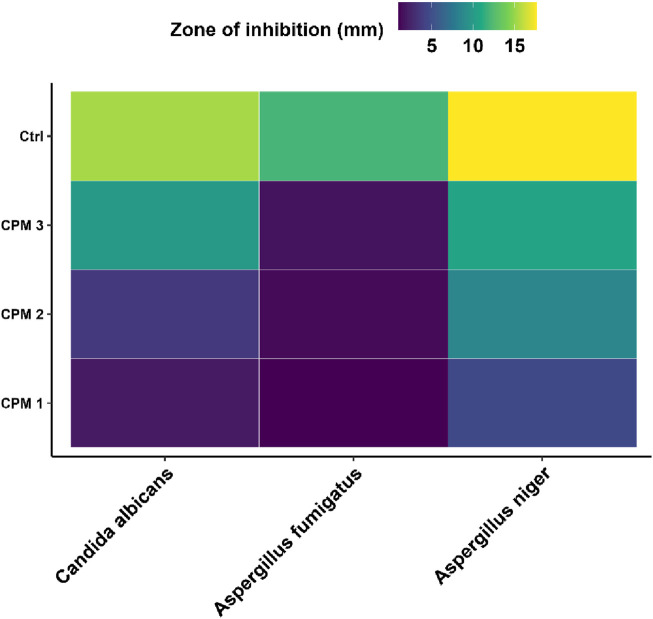
Heatmap visualization of antifungal efficacy of CPM against fungal pathogens.

Heatmaps were constructed to visually represent/compare the antimicrobial activity of different solvents across bacterial strains Figures 12, 13. These heatmaps vividly capture the concentration-dependent, selective antimicrobial efficacy of the tested phytocompound mixtures.

Molecular dynamics validation further reinforces these findings. Our equilibrated RMSD values (1.2–1.5 Å for protein–ligand complexes) and low RMSF (0.69–1.07 Å) indicate greater stability than typical flavonoid enzyme systems and align closely with Ramalingam et al. (2023), who reported RMSD ∼2 Å and Rg ∼1.54 nm over 10 ns for carpachromene–KRAS complexes. In a more recent virtual screening study, Herlina et al. (2025) ranked carpachromene among 473 Erythrina flavonoids against SARS-CoV-2 RdRp (37th position) and noted favorable ADMET properties (including BBB permeability), underscoring its drug-like profile. Notably, our 100 ns simulations extend these observations by confirming stable Rg (∼16.5 Å), SASA (∼9300 Å^2^), and positive DCCM/PCA correlations metrics absent in prior carpachromene studies, thus providing the first evidence of multi-target microbial engagement without dissociation.

These computational insights mechanistically explain the potent *in-vitro* antimicrobial activity (21 mm zone against *S. enterica*; 10 mm against *C. albicans*), which exceeds or matches standard controls. This multi-target profile positions carpachromene as a promising AMR-evading scaffold, particularly given rising resistance to sulfonamides, quinolones, and azoles. Future optimization via supradecoration could further enhance potency, as suggested by analogous flavonoid SAR studies.

## Conclusion

The integrated *in-silico* and *in-vitro* investigation of carpachromene, a prenylated chromene derivative from *V*. *thapsus* signifies compelling evidence towards its broad-spectrum antimicrobial propensity. In conclusion, carpachromene emerges as a promising natural scaffold with multi-target antimicrobial properties, offering a valuable lead for the development of novel therapeutic agents. Comprehensive molecular docking coupled with 100 ns molecular dynamics simulations of carpachromene with multiple microbial protein targets have added to the computational methodologies in antimicrobial drug development. The evaluated descriptors of chemico-biological interaction of carpachromene with selected microbial protein targets: CYP51, dihydropteroate synthase, GyrB ATPase, and PBP-1, demonstrated stable and specific binding interactions depicting molecular level mechanistic insights valuable in designing multi-target antimicrobial drugs. The docking and simulation findings have been substantiated by *in-vitro* assays corroborating concentration-dependent antimicrobial activity against selected pathogenic strains.

## Data Availability

The original contributions presented in the study are included in the article/Supplementary Material, further inquiries can be directed to the corresponding authors.
