# Supplementary material for: Chemico-biological evaluation of carpachromene against key antimicrobial protein targets: an integrated in-silico, in-vitro approach for mechanistic insights
Source: Front Pharmacol. 2026 Apr 17;17:1785267. doi: 10.3389/fphar.2026.1785267 (PMC13133430; doi:10.3389/fphar.2026.1785267)
Supplement: Supplementary file 1 [file Supplementaryfile1.docx]

| **Table S1.** 2-way Anova table ZOI ~ Treatment x Bacteria | | | | | |
| --- | --- | --- | --- | --- | --- |
| Term | *df* | sumsq | meansq | F value | *p* |
| Extract | 3 | 2,370.82 | 790.27 | 18,966.56 | < .001*** |
| Bacteria | 5 | 1,671.13 | 334.23 | 8,021.40 | < .001*** |
| Extract × Bacteria | 15 | 298.93 | 19.93 | 478.29 | < .001*** |
| Residuals | 48 | 2.00 | 0.04 |  |  |

| **Bacteria** | **Treatment** | **Mean** | ***SE*** | **cld** |
| --- | --- | --- | --- | --- |
| *S. aureus* | Ctrl | 14.33 | 0.33 | a |
| *S. aureus* | CPM 3 | 9.00 | 0.00 | b |
| *S. aureus* | CPM 1 | 1.00 | 0.00 | c |
| *S. aureus* | CPM 2 | 1.00 | 0.00 | c |
| *P. aeroginosa* | Ctrl | 20.00 | 0.00 | a |
| *P. aeroginosa* | CPM 3 | 10.00 | 0.00 | b |
| *P. aeroginosa* | CPM 1 | 1.00 | 0.00 | c |
| *P. aeroginosa* | CPM 2 | 1.00 | 0.00 | c |
| *E. coli* | Ctrl | 20.00 | 0.00 | a |
| *E. coli* | CPM 3 | 13.00 | 0.00 | b |
| *E. coli* | CPM 2 | 10.00 | 0.00 | c |
| *E. coli* | CPM 1 | 2.00 | 0.00 | d |
| *B.cereus* | Ctrl | 15.33 | 0.33 | a |
| *B.cereus* | CPM 3 | 11.00 | 0.00 | b |
| *B.cereus* | CPM 2 | 3.00 | 0.00 | c |
| *B.cereus* | CPM 1 | 1.00 | 0.00 | d |
| *K. pneumoniae* | Ctrl | 20.00 | 0.00 | a |
| *K. pneumoniae* | CPM 3 | 11.00 | 0.00 | b |
| *K. pneumoniae* | CPM 2 | 9.00 | 0.00 | c |
| *K. pneumoniae* | CPM 1 | 1.00 | 0.00 | d |
| *S. enterica* | Ctrl | 25.00 | 0.00 | a |
| *S. enterica* | CPM 3 | 21.00 | 0.00 | b |
| *S. enterica* | CPM 2 | 20.00 | 0.00 | c |
| *S. enterica* | CPM 1 | 17.33 | 0.33 | d |

| **Table S2.** 2-way Anova table ZOI ~ Treatment x Fungi | | | | | |
| --- | --- | --- | --- | --- | --- |
| Term | *df* | sumsq | meansq | F value | *p* |
| Treatment | 3 | 803.89 | 267.96 | 876.97 | < .001*** |
| Fungi | 2 | 249.39 | 124.69 | 408.09 | < .001*** |
| Extract × Fungi | 6 | 55.94 | 9.32 | 30.52 | < .001*** |
| Residuals | 24 | 7.33 | 0.31 |  |  |

| Fungi | Treatment | Mean | *SE* | cld |
| --- | --- | --- | --- | --- |
| *Candida albicans* | Ctrl | 15.33 | 0.33 | a |
| *Candida albicans* | CPM 3 | 10.00 | 0.00 | b |
| *Candida albicans* | CPM 2 | 3.67 | 0.33 | c |
| *Candida albicans* | CPM 1 | 2.00 | 0.00 | d |
| *Aspergillus fumigatus* | Ctrl | 12.00 | 0.58 | a |
| *Aspergillus fumigatus* | CPM 3 | 1.67 | 0.33 | b |
| *Aspergillus fumigatus* | CPM 2 | 1.33 | 0.33 | b |
| *Aspergillus fumigatus* | CPM 1 | 1.00 | 0.00 | b |
| *Aspergillus niger* | Ctrl | 17.67 | 0.33 | a |
| *Aspergillus niger* | CPM 3 | 10.67 | 0.33 | b |
| *Aspergillus niger* | CPM 2 | 8.67 | 0.33 | c |
| *Aspergillus niger* | CPM 1 | 4.67 | 0.33 | d |

**Figure S1.** FTIR spectrum of the carpachromene compound


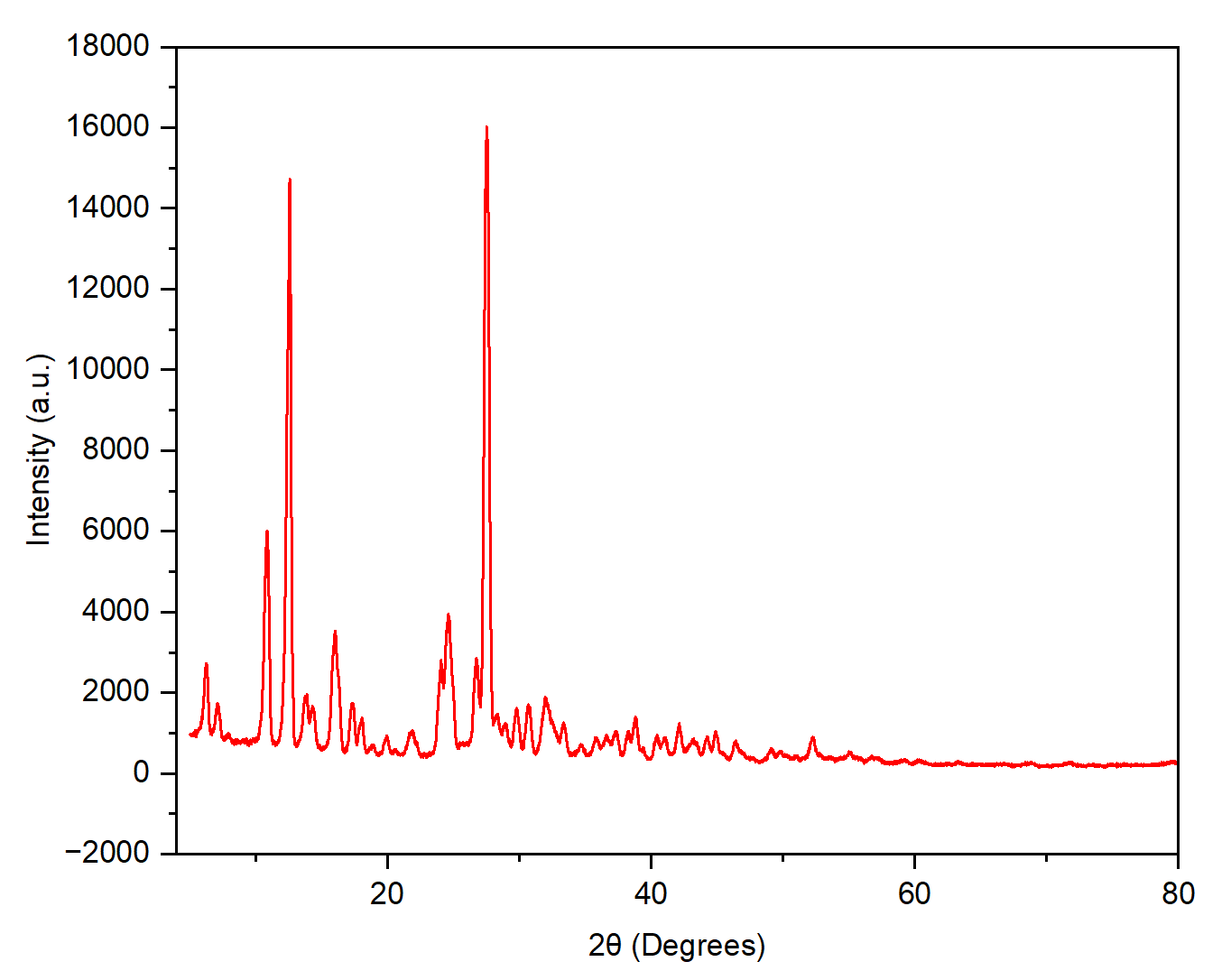
 **Figure S2.** X-ray diffraction (XRD) pattern of the isolated compound

**^1^H NMR** (400 MHz, DMSO d_6_) δ 7.45 – 7.38 (m, 2H, Ar-Region), 6.96 – 6.82 (m, 2H, Ar-Region), 6.59 (1H, Doublet, Alkene), 6.30 (s, 1H, Alkene), 6.23(s, 1H, Ar-Region), 6.17 (1H, Doublet, Alkene), 1.56 (s, 6H).

(Rest all peaks are impurities and solvent DMSO at 2.5 ppm)

**
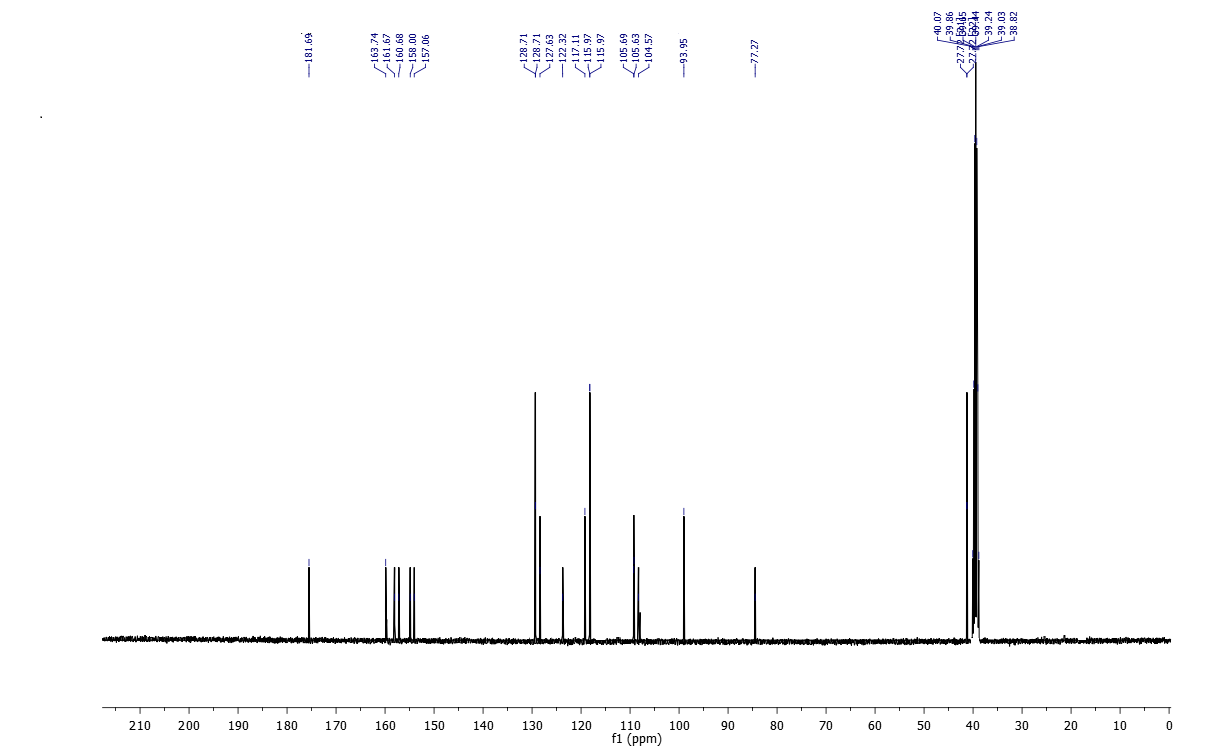
**

**^13^C NMR** (101 DMSO d6) δ 181.69 (s), 163.74 (s), 161.67 (s), 160.68 (s), 158.00 (s), 157.06 (s), 128.91 – 128.51 (m), 127.63 (s), 122.32 (s), 117.11 (s), 116.07 – 115.86 (m), 105.66 (d, J = 8.1 Hz), 104.57 (s), 93.95 (s), 77.27 (s), 27.82 – 27.51 (m)

**Figure S3.** ^1^H NMR ^13^C NMR of the isolated compound

A)


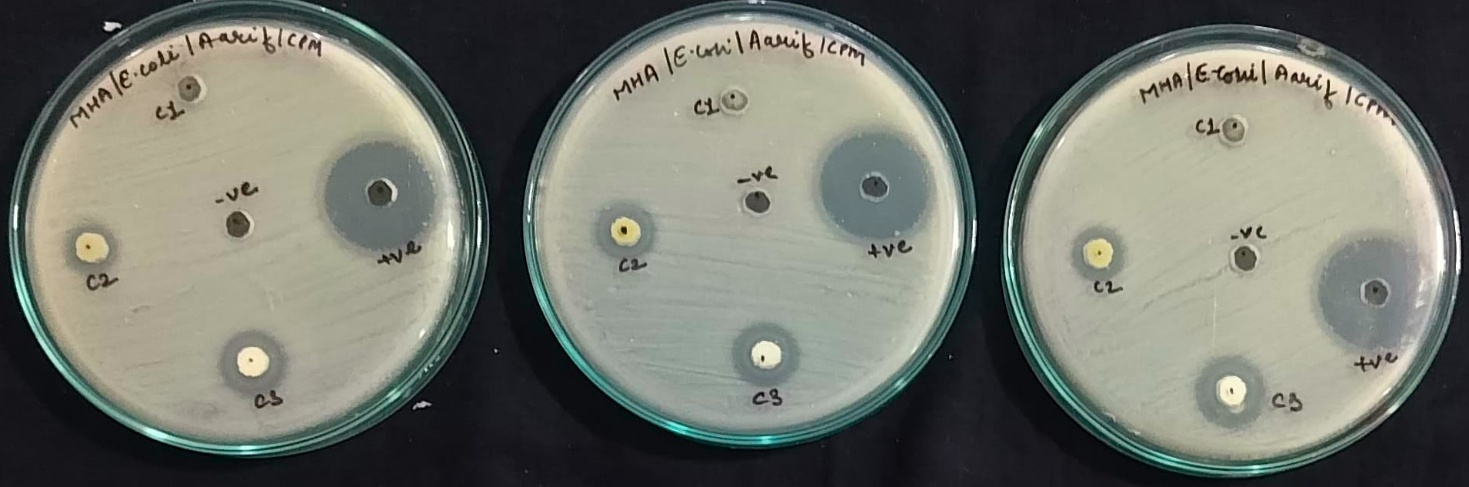


B)


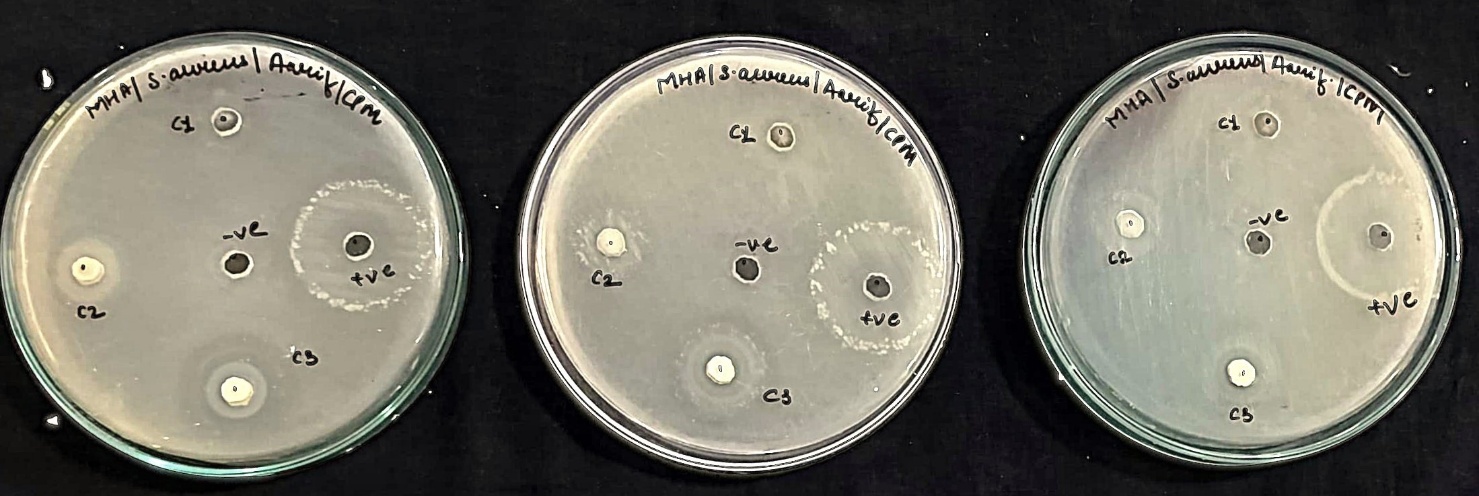


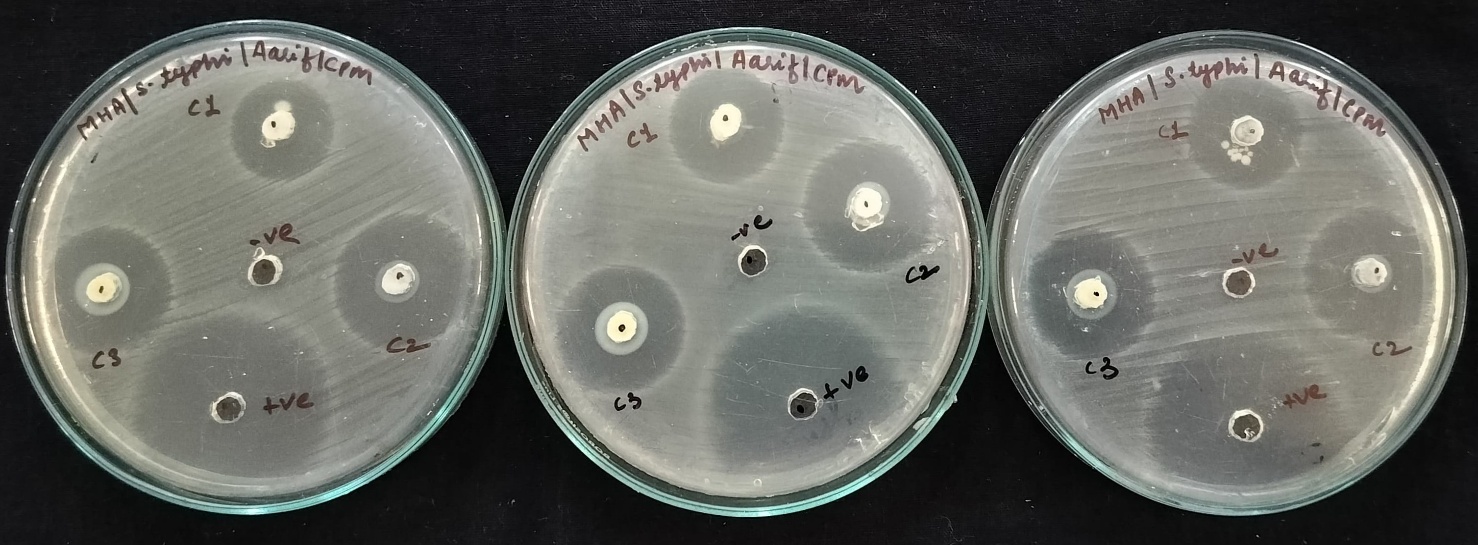


C)


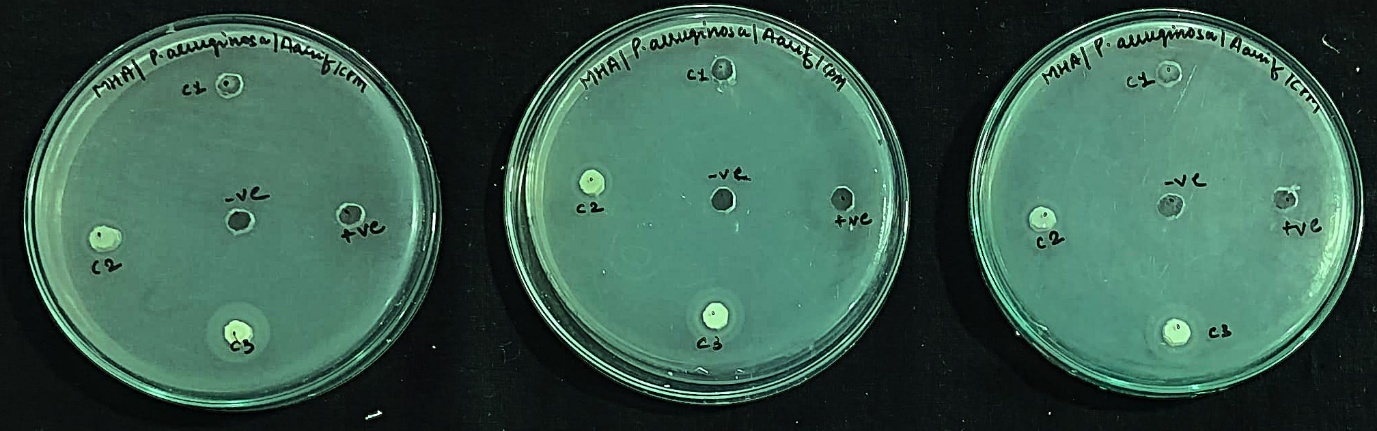


E)

D)


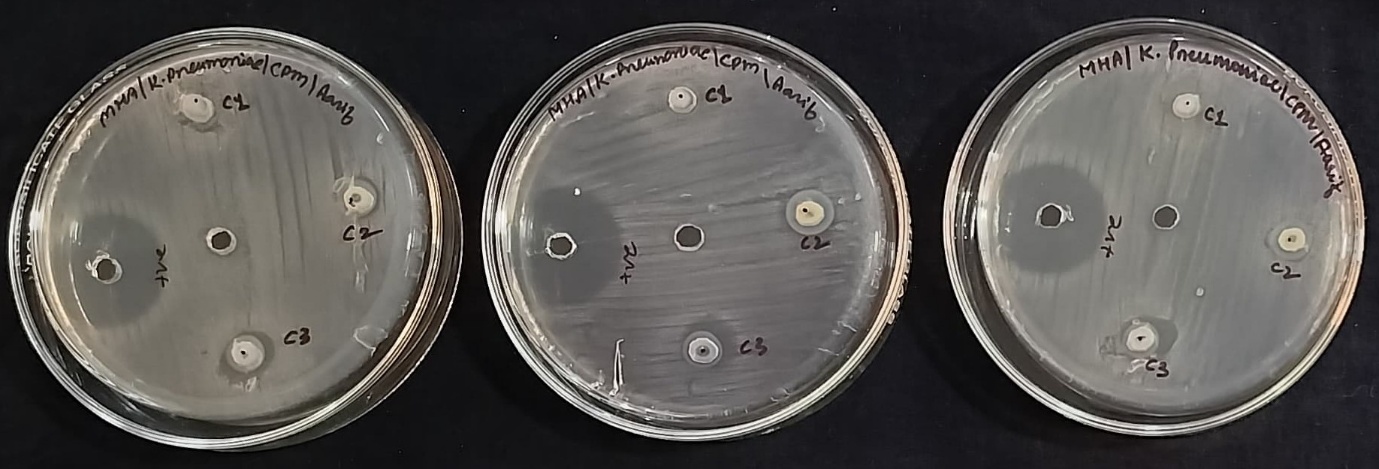


F)


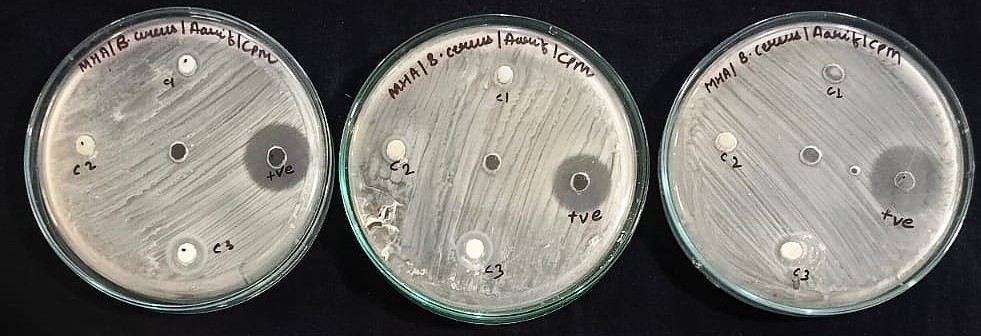


G)


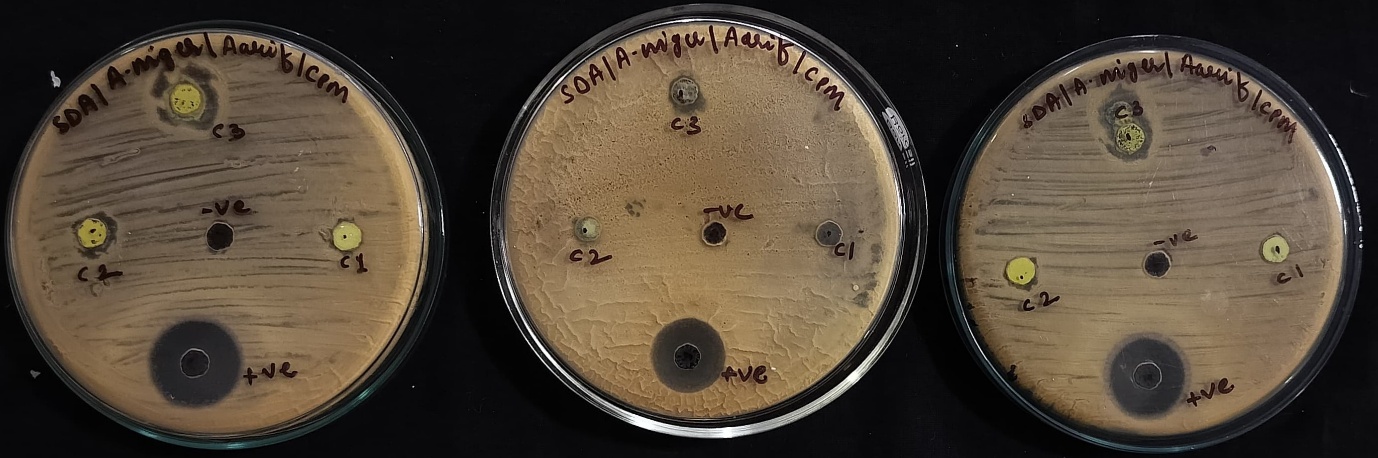


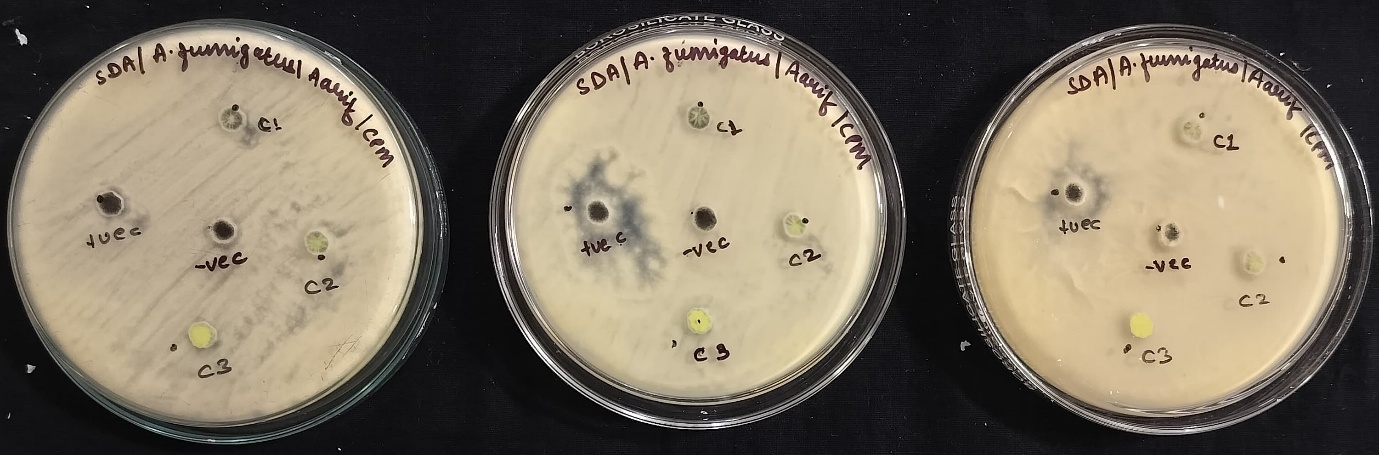


I)

H)


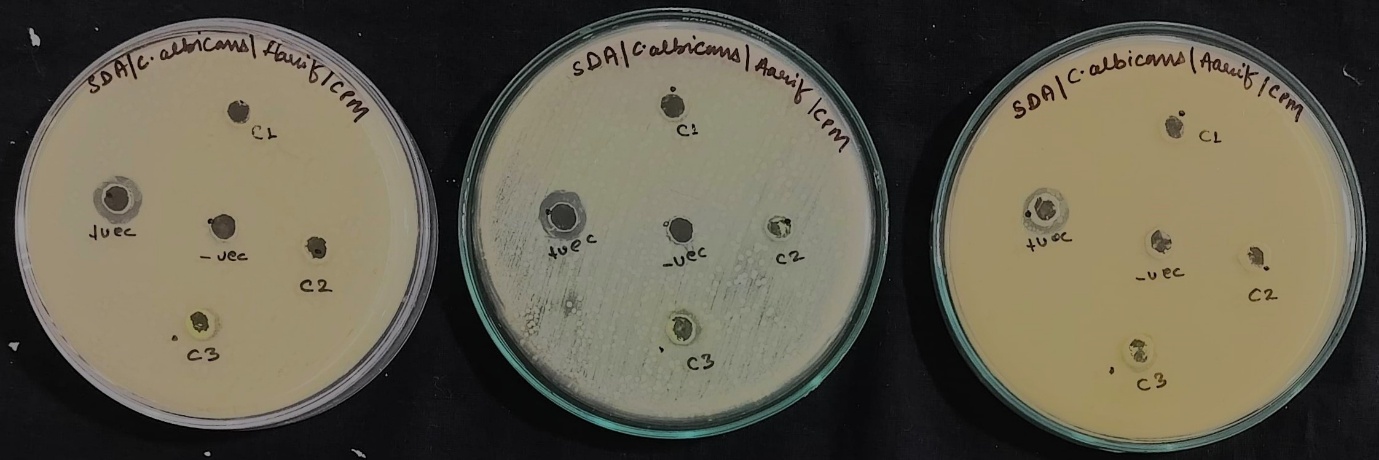


**Figure S4.** Antibacterial activity of the carpachromene compound against different **bacterial** pathogens: *(A). Escherichia coli (B). Staphylococcus aureus (C). Salmonella enterica (D). Pseudomonas aeruginosa (E). Klebsiella pneumoniae (F). Bacillus cereus) and* **fungal** *(G). Aspergillus niger (H). A. fumigatus (I). Candida albicans)*
